# Putting the “learning” in “pre-learning”: effects of a self-directed study hall on skill acquisition in a simulation-based central line insertion course

**DOI:** 10.1186/s41077-023-00261-4

**Published:** 2023-09-08

**Authors:** Emily Diederich, Matthew Lineberry, Vanessa Schott, Julie Broski, Ahmed Alsayer, Krista A. Eckels, Megan J. Murray, William Huynh, Laura A. Thomas

**Affiliations:** 1 Division of Pulmonary and Critical Care Medicine, Department of Medicine, University of Kansas School of Medicine, Kansas City, KS USA; 2 Zamierowski Institute for Experiential Learning, University of Kansas Medical Center and Health System, Kansas City, KS USA; 3 Department of Population Health, University of Kansas School of Medicine, Kansas City, KS USA; 4 Veteran’s Affairs Eastern Kansas Health Care System, Topeka, KS USA; 5 Department of Surgery, University of Kansas School of Medicine, Kansas City, KS USA; 6 College of Science and Arts, Taibah University, Almadinah Almunawwarah City, Kingdom of Saudi Arabia; 7 Department of Occupational Therapy, School of Health Professions, Kansas City, KS USA; 8 University of Kansas School of Medicine, Kansas City, KS USA

**Keywords:** Pre-learning, Simulation, Self-directed learning, Central line insertion, Procedural training

## Abstract

**Background:**

Opportunities to practice procedural skills in the clinical learning environment are decreasing, and faculty time to coach skills is limited, even in simulation-based training. Self-directed learning with hands-on practice early in a procedural skill course might help maximize the benefit of later faculty coaching and clinical experience. However, it may also lead to well-learned errors if learners lack critical guidance. The present study sought to investigate the effects of a hands-on, self-directed “study hall” for central line insertion among first-year residents.

**Methods:**

Learner cohorts before vs. after introduction of the study hall (*n* = 49) were compared on their pre- and post-test performance of key procedural behaviors that were comparable across cohorts, with all learners receiving traditional instructor-led training between tests.

**Results:**

Study hall participants spent a median of 116 min in hands-on practice (range 57–175). They scored higher at pre-test (44% vs. 27%, *p* = .00; Cohen’s *d* = 0.95) and at post-test (80% vs. 72%, *p* = .02; Cohen’s *d* = 0.69). A dose–response relationship was found, such that 2 h of study hall were roughly equivalent to the performance improvement seen with four clinical observations or supervised insertions of central lines.

**Conclusions:**

Self-directed, hands-on “study hall” supported improved procedural skill learning in the context of limited faculty availability. Potential additional benefits make the approach worth further experimentation and evaluation.

**Supplementary Information:**

The online version contains supplementary material available at 10.1186/s41077-023-00261-4.

## Background

As the Accreditation Council for Graduate Medical Education and others are pushing for more objective measurement of learners’ preparedness for practice in healthcare [1], opportunities to focus on the learning and maintenance of procedural skills are under pressure from multiple directions. Learners needing practice opportunities are finding that procedures are increasingly being performed by specialized proceduralist services and prehospital providers [2] or are becoming more rare given alternative treatments that may be provided [3]. Meanwhile, the increasing patient volume and pace of the clinical environment makes it more challenging than ever for faculty and learners to set aside time for procedural instruction while in the workplace [4]. It should thus not be surprising that learners often struggle to perform key procedures [5, 6]. Simulation is a powerful tool for learning [7], though simulation also requires the scarce time of faculty for the instruction, as well as professional development related to simulation-based education.

Ideally, learning resources could help learners “move up” the learning curve more quickly, using faculty time only when it is most necessary. For instance, central line insertion is a complex procedure, but it is not clear that all aspects of it require expert instruction. Some learning involves simply orienting to the vast array of necessary equipment or practicing unfamiliar but straightforward maneuvers. If learners could gain foundational knowledge and skill beforehand, time with instructors could be focused on task components not amenable to self-directed learning — perhaps finer points of ultrasound/needle coordination, for instance. Simulation-based procedural training courses do often feature “pre-learning” assignments, such as journal article readings or multimedia lectures and demonstrations [8, 9]. However, as these are often relatively passive, they seem unlikely to be highly effective or well-retained [10]. Far less common, and somewhat controversial, would be to support learners in self-directed procedural practice during the “pre-learning” phase.

Learning science is unclear as to whether such early self-directed practice by novice learners enhances or inhibits learning. Several theories, including cognitive load theory, encourage maximum coaching early in the learning process [11, 12]. Findings also suggest that learners struggle to self-assess their learning [13] and thus may make poor use of independent practice opportunities. Consistent with this, a recent study found that expert feedback during early deliberate practice supported greater learning of endourologic skills than did feedback provided during a later deliberate practice session [14]. However, other frameworks encourage educators to provide more self-directed learning opportunities [15], and preliminary evidence suggests that for fundamental laparoscopic skills, learner self-directed practice on take-home “box trainers” has led to positive learning outcomes with minimal prior coaching [16]. As such, we wondered how incorporating more self-directed practice early in a procedural skill course would affect learning.

In this study, we investigated residents’ scores on simulation-based central line insertion assessments conducted both before and after a traditional instructor-led training session, comparing learners who did versus did not attend an initial self-directed practice “study hall.” We hypothesized that study hall would facilitate higher pre-test scores and greater learning gains from instructor-led training.

## Methods

The study is quasi-experimental, with pre- and post-test across two cohorts of first-year internal medicine residents from subsequent academic years. The initial cohort served as the control group for the following academic year in which treatment group residents participated in the study hall intervention prior to the three curricular components common to both groups: pre-test, instructor-led training, and post-test. The study was approved by the University of Kansas Medical Center Institutional Review Board.

### Participants

Learner demographics are given in Table 1. Forty-nine of 51 learners consented to participate, all being first-year internal medicine residents in a mandatory central line insertion course. We focused analyses on first-year residents since performance scores for more senior residents would be confounded with prior simulation-based training experiences.

**Table 1:** Participant demographics

	AY 2016	AY 2017
Number of participants	24	25
Number of MICU rotation months		
0	59%	32%
1	41%	44%
2	0%	24%
Number of central line insertions observed		
0	46%	33%
1	36%	21%
2	18%	17%
3	0%	17%
4	0%	8%
5	0%	4%
Number of supervised central line insertions		
0	86%	52%
1	9%	48%
2	0%	0%
3	5%	0%

*AY* Academic year, *MICU* Medical intensive care unit

### Measures

Each learner completed two central line insertion assessments (pre- and post-test) in a simulated environment. During the assessment, each learner had the opportunity to place a central line on a manikin situated in a simulated hospital room staged with the equipment and supplies identical to those found in the local clinical environment. Study personnel served as the patient voice, and a chief resident was trained to play the role of non-sterile assistant, which enabled learners to attempt a proper insertion from patient greeting through final assurance of successful insertion. After each pre- and post-test, the chief resident shared their observations related to errors with the learner in a debrief. Video and audio recordings were reviewed by trained observers using a scoring key, which was designed by an interdisciplinary group of expert clinicians for the local health system, including procedural steps and associated observable behaviors for each (Additional file 1: Appendix A). To mitigate internal validity threats associated with quasi-experimental research, the research team convened to compare assessment and training particulars across the two cohorts, noting any inconsistencies that might compromise fair comparison. For this study, we reduced the data down to comparable behaviors only. A sample of 20% of assessments were double coded to ensure reliability.

Additionally, a demographic survey was administered to all participants and included self-reported number of central lines inserted and observed prior to study hall. Videos of each learner’s journey through their study hall session were reviewed by the research team. The time spent in hands-on practice was recorded as the time between completion of the study hall orientation activities and the initiation of the exit survey.

### Study procedures

There was guidance provided to support the self-directed practice during the study hall sessions. A nonclinical proctor provided an orientation to the individual learning stations including a simulator, ultrasound, line insertion equipment, and iPad with a multimedia didactic and demonstration learning module. Learners were provided with a list of procedural steps, each of which was demonstrated in the institution-specific videos within the learning module. Between one and four learners attended for any given session and were free to learn separately or collaboratively. The proctor was present for the session to answer basic questions and address equipment issues. Participants were encouraged to practice for at least 2 h but were free to practice longer as desired and feasible.

Approximately, 4 weeks later (or for control participants, as their initial course experience), participants completed the pre-test. Later that week, they completed an approximately 2-h instructor-led training session with two faculty instructors and up to five learners. Finally, again later that week, they completed the post-test.

### Analyses

We used generalizability theory to estimate measurement reliability and then compared pre- and post-test scores by condition to estimate the overall effect of the intervention. To investigate dose–response relationships, pre-test scores were regressed on study hall time as the main predictor of interest, controlling for each participant’s number of central line insertions either observed or performed under supervision in clinical practice. Post-test scores were similarly regressed on study hall time and number of observed or supervised insertions, along with pre-test scores. Finally, we explored which items showed the greatest differences in probability of correct performance between control and treatment participants.

## Results

### Descriptive statistics

Among the treatment group, omitting the three participants unable to attend, median time spent in hands-on practice during study hall was 116 min, ranging from 57 to 175 min.

### Reliability

Generalizability analyses of double-coded scores (within a fully crossed, learners-by-items-by-raters model, with items fixed) showed rank-order reliability of 0.83 for an individual item and 0.97 for total scores.

### Treatment–control differences

**Fig. 1:**
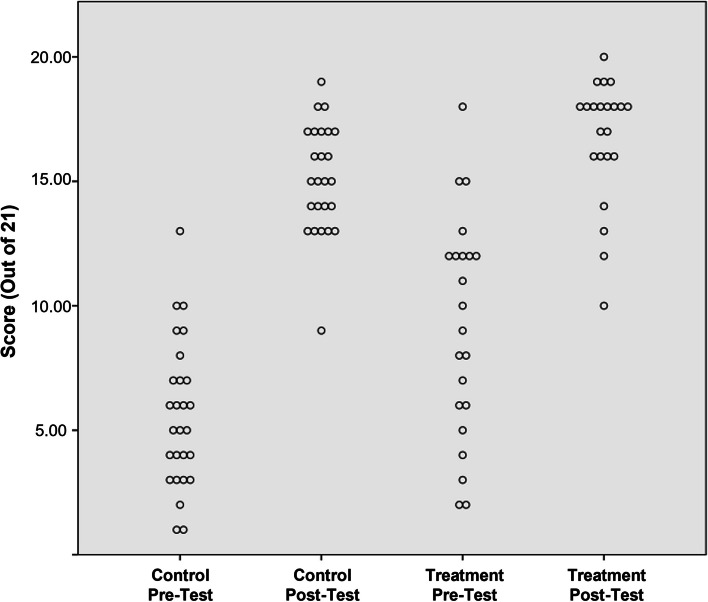
Total correct scores on procedural steps at pre- and post-test, control vs. treatment

We found statistically significant differences between the control and treatment groups in both the pre-test and the post-test scores. First, Fig. 1 depicts the discernible difference in pre-test scores for learners in the control vs. treatment groups (unequal variance *t* = 3.25, *p* = .00). Mean control and treatment group scores were 27% and 44%, respectively (*SDs* = 14% and 21%), resulting in a large effect size of the study hall intervention (Cohen’s *d* = 0.95). Second, a discernible effect was also seen for post-test scores (*t* = 2.35, *p* = .02). Control and treatment group means were 72% and 80%, respectively (*SDs* = 10% and 12%), resulting in a medium effect size (Cohen’s *d* = 0.69).

### Dose–response effects: controlling for insertion experience

In the regression model predicting pre-test scores, minutes spent in study hall was a significant predictor (*p* = .03), while number of observed or supervised insertions was not (*p* = .09). The predicted score on pre-test for an average learner not attending study hall was 25%. Spending 120 min in study hall was predicted to improve that score by 12 percentage points (to 37%), roughly equivalent to the improvement predicted from four observations or supervised insertions in the clinical environment. In the regression model predicting post-test scores, only pre-test scores were a significant predictor (*b*_pretest_ = 0.32, *p* = .00). For both regression models, diagnostics were favorable (i.e., significant model fit and low variance inflation factors).

### Item analyses

Table 2 shows percentage of learners who completed each step correctly on the pre-test and post-test for both the control and treatment groups, along with “normalized gain or loss” for each item [17]. The normalized gain quantifies the proportion of the possible gain the treatment group achieved relative to how much room for improvement there was in the control group performance. For example, referring to Table 2, 11% of learners in the control group completed “prepare insertion kit” correctly on the pretest. The maximal amount of gain, or improvement, possible for the treatment group would be an additional 89% (i.e., 100–11% = 89%). In actuality, 59% of the learners in the treatment group completed the step correctly on the pre-test after having participated in study hall. Thus, the normalized gain of the treatment group compared with the control group for this item was 54% (i.e., (59–11%)/89%)). All but two of 21 items showed a normalized gain on the pre-test in learners who participated in the study hall.

**Table 2:** Item analysis. Percent of learners correct by procedural step: control vs. treatment at pre- and post-test

Procedural step	Pre-test			Post-test		
	Control	Treatment	Normalized gain or loss (% of possible)	Control	Treatment	Normalized gain or loss (% of possible)
01. Administer informed consent	100%	91%	− 9%	100%	100%	0%
02. Lead time-out	7%	18%	12%	100%	68%	− 32%
03. Don hat and mask	74%	77%	12%	72%	100%	100%
04. Unpack bundle and line kit	63%	41%	− 35%	80%	95%	75%
05. Clean area with chlorhexidine	11%	45%	38%	52%	73%	44%
06. Don gown and gloves	22%	36%	18%	64%	77%	36%
07. Prepare insertion kit	11%	59%	54%	20%	91%	89%
08. Apply sterile drape	7%	36%	31%	56%	55%	− 2%
09. Apply sterile sheath to ultrasound probe	37%	64%	43%	72%	82%	36%
10. Set bed to Trendelenburg	44%	45%	2%	64%	86%	61%
11. Inject lidocaine	26%	45%	26%	72%	77%	18%
12. Advance seeker needle with ultrasound	30%	45%	21%	96%	82%	− 15%
13. Acquire venous return	44%	50%	11%	92%	82%	− 11%
14. Confirm guidewire with ultrasound	0%	27%	27%	44%	68%	43%
15. Knick skin	11%	23%	13%	88%	82%	− 7%
16. Dilate vein	19%	41%	27%	96%	91%	− 5%
17. Advance catheter	15%	41%	31%	76%	86%	42%
18. Remove guidewire	4%	45%	43%	76%	82%	25%
19. Flush ports	0%	5%	5%	28%	27%	− 4%
20. Secure and dress catheter	11%	41%	34%	80%	82%	10%
21. Order chest X-ray	22%	41%	24%	84%	86%	13%

If treatment > control, “treatment improvement (% of possible)” = (treatment — control)/(100% — control). If treatment < control, “treatment decrement (% of possible)” = (treatment — control)/(control)

## Discussion

This study investigated the effects of a self-directed, hands-on “study hall” for central line insertion. Consistent with hypotheses, study hall participation was associated with considerable gains in both pre- and post-test scores. Regression analyses suggest that the effects persist even after controlling for line insertion experiences in the clinical environment, in the form of enhanced pre-test scores, which then predict enhanced post-test scores. The effect sizes compare favorably to learning associated with observation and supervised insertion in the clinical learning environment, suggesting the approach may be used to complement clinical experience.

One motivating factor in the design of the study hall intervention was to facilitate self-directed learning for more basic procedural steps that may be accomplished without the benefit of expert coaching, thereby preserving time with faculty for deliberate practice of more advanced skills. Although comparative analysis of the performance on individual procedural steps between the control and treatment groups is beyond the scope of this study, we did calculate the normalized gain (or loss), which is the proportion of the possible gain the treatment group achieved relative to how much room for improvement there was in the control group performance. The magnitude of the normalized gain was most positive for several basic procedural steps (e.g., prepare insertion kit, 54% of possible improvement at pretest, 89% at posttest; clean area with chlorhexidine, 38% and 44% of possible improvement respectively). This supports our vision for the progression of learning at each phase: that “study hall” offers ample amounts of practice for any content that does not require close instruction or coaching, formal training ensures sufficient (but necessarily limited) time for deliberate practice with expert feedback on more difficult-to-learn content, and then the clinical learning environment — obviously the most realistic but also least amenable to learner-adaptive deliberate practice — is where learners master the most complex task elements (e.g., patient variation).

One possible risk of self-directed, hands-on practice is that novices learn incorrect procedures. We did not see evidence of such negative learning overall in this investigation, as mean treatment group scores on both the pre- and post-tests were higher than the mean control group scores, and that difference was statistically significant. When looking at performance across individual procedural steps in Table 2, the average treatment group score was greater than the average control group for 19/21 of the procedural steps in the pre-test and for 13/21 on the posttest. Further exploration of the performance variation in normalized gain or loss across the procedural steps is an area ripe for further investigation.

Several aspects of this intervention make us optimistic; it can show even greater positive learning effects. For one, this was the first offering of study hall for our institute, and we have since refined aspects of it to encourage more effective peer learning practices [18] and more use of assessment for learning [19], consistent with the finding that guided self-direction is more effective than both unstructured self-direction and non-learner-directed practice [20]. Additionally, the study hall sessions include first-, second-, and third-year residents which has led to very impactful peer coaching, particularly when senior residents are paired with those with less experience. Since learners partially structure their own practice, study hall also creates opportunities for feedback and coaching on learners’ self-regulated learning strategies, referred to as “second-order scaffolding”[21] or “preparation for future learning” [22]. For instance, an educator might prompt reflection when a learner opts to engage in little practice and then shows sub-par performance later or an educator might cheer the fact that a learner strove to push themselves and make productive errors that they then learned from [23]. Finally, the intervention is scalable and convenient to learners. While the equipment required is substantial, it may be provided in a room with minimal support, without schedules needing to be aligned between learners and faculty. Study hall also makes it easier for learners to space out practice over multiple sessions, which can dramatically improve learning [24, 25].

The quasi-experimental nature of the study limits inferences somewhat, though we applied several statistical and logical controls to reduce validity threats [26]. Controlling for prior exposure to the central line course meant that we only investigated the study hall’s effects with first-year residents; however, we offer study hall to more senior residents as well, and it would be interesting to gauge its effects on their learning, to the extent that such effects can be disentangled from other factors related to their performance.

Several lines of follow-on research seem promising to us. First, it would be useful to model and maximize learner engagement in self-directed practice and to optimize learner’s practice strategies. One “high-tech” possibility in development is the use of computer-intelligent sensing and tutoring, to partially play the role a live coach might play in guiding learners [27, 28]. Similarly, “low-tech” peer coaching might help ensure more effective practice. Second, we are curious what the curricular impacts might be of adopting more guided self-directed learning of this nature — specifically, whether it improves future learning behaviors broadly, outside of the simulation center and/or beyond the specific procedure being learned. Third, we are interested to explore the impact of self-directed, hands-on learning on the durability of learning, as well as experiment with the impact of self-directed learning as a follow-up to instructor led instruction.

## Conclusions

Our initial evaluation of a self-directed “study hall” with high-fidelity practice opportunities for central line insertion suggests the approach can have powerful effects for learning. As the practice of simulation-based healthcare education grows, we anticipate that guided learner self-direction will play an increasing role in helping expand simulation’s reach and impact.

### Supplementary Information

**Additional file 1: Appendix A.** Central line insertion assessment: Concordance of comparable behaviors and procedural steps for academic year 2016 vs. 2017.

## Data Availability

The datasets used and/or analyzed for the current study are available from the corresponding author on reasonable request.
