# Supplementary material for: Putting the “learning” in “pre-learning”: effects of a self-directed study hall on skill acquisition in a simulation-based central line insertion course
Source: Adv Simul (Lond). 2023 Sep 8;8:21. doi: 10.1186/s41077-023-00261-4 (PMC10486059; doi:10.1186/s41077-023-00261-4)
Supplement: Supplementary file 1 — Additional file 1: Appendix A. Central line insertion assessment: Concordance of comparable behaviors and procedural steps for academic year 2016 vs. 2017. [file 41077_2023_261_MOESM1_ESM.pdf]

Appendix A

Central line insertion assessment: Concordance of comparable behaviors and procedural steps for academic year 2016 vs. 2017

KU Health System CVC Insertion Procedural Steps		**Behavior to Demonstrate**	**Suitable for comparison**	**Reason if not comparable**	**Scoring rule**
1. Select Kit	1	**Asks for 16cm line kit**	No	Residents didn't select line lengths in AY16	Feedback
2. Wash Hands	2	**Cleans their hands with alcohol hand rub before contact with the patient**	No	May have done before entering the room in AY16	Point lost
3. Obtain Informed Consent	3a	**Explains important aspects of the procedure without jargon**	No	Not part of AY16 training	Feedback
	3b	**Explains at least three major risks**	Yes		Point lost
	3c	**Explains at least one benefit/necessity for the procedure**	No	Not part of AY16 training	Point lost
	3d	**Explains the alternative(s)**	No	Not part of AY16 training	Feedback
4. Conduct Timeout	4a	**Confirms at least two patient identifiers**	Yes		Point lost
	4b	**Confirms the procedure to be performed**	Yes		Point lost
	4c	**Confirms the site**	No	Not part of AY16 training	Point lost
	4d	**Confirms at least one aspect of relevant medical histor**y	No	Not part of AY16 training	Feedback
5. Identify Target Area Using Ultrasound	5a	**Verifies correct probe orientation physically**	No	Not part of AY16 training	Point lost
	5b	**Orients the probe correctly**	No	Poor US visibility in AY16 videos	Point lost
	5c	**Compresses the vessels and moves the probe down the neck**	No	Not part of AY16 training	Point lost
	5d	**Sets an appropriate depth**	No	Poor US visibility in AY16 videos	Point lost
	5e	**Sets an appropriate gain level and/or uses sufficient gel**	No	Poor US visibility in AY16 videos	Point lost
	5f	**Wipes gel off neck**	No	Not part of AY16 training	Feedback
6. Don Hat and Mask	6a	**Dons hat** Before unpacking sterile contents OR applying chlorhexidine	Yes		Retest
	6b	**Dons mask** Before unpacking sterile contents of bundle, OR before applying chlorhexidine	Yes		Retest
	7a	**Throws first layer of bundle wrapping away**	No	Not part of AY16 training	Feedback
	7b	**Unfolds inner wrap of bundle in a sterile fashion**	Yes		Retest

KU Health System CVC Insertion Procedural Steps		**Behavior to Demonstrate**	**Suitable for comparison**	**Reason if not comparable**	**Scoring rule**
7. Unpack Bundle and Line Kit in Sterile Fashion	7c	**Places sterile syringes, ultrasound cover, & gloves on the sterile field in a sterile fashion**	Yes		Retest
	7d	**Opens the line kit in a sterile fashion or asks nurse to do so**	Yes		Retest
8. Clean Area with Chlorhexidine	8a	**Cleans with chlorhexidine for 30+ seconds prior to draping the patient**	Yes		Point lost
	8b	**Scrubs vigorously back-and-forth over at least a 4"x5" area**	Yes		Point lost
	8c	**Allows the area to dry 30+ seconds before draping patient**	Yes		Point lost
9. Don Gown and Gloves	9a	**Dons their gown in a sterile fashio**n	Yes		Retest
	9b	**Dons their gloves in a sterile fashion**	Yes		Retest
10. Prepare Insertion Kit	10a	**Prepares the lidocaine**	Yes		Point lost
	10b	**Checks the seeker needle**	No	Not part of AY16 training	Feedback
	10c	**Uncaps, advances, and retracts the guidewire**	Yes		Feedback
	10d	**Opens and closes the scalpel**	No	Not part of AY16 training	Feedback
	10e	**Removes the brown port cap before advancing the guidewire**	No	Not part of AY16 training	Feedback
	10f	**Flushes the line and ports**	No	Not part of AY16 training	Point lost
11. Drape Patient	11	**Properly aligns and applies the sterile drape to the patient**	Yes		Retest
12. Place Sterile Sheath and Gel on Ultrasound Probe	12a	**Places gel on the probe in a sterile fashion prior to sheathin**g or asks the nurse to do this	Yes		Retest
	12b	**Sheathes and secures probe in a sterile fashion**	Yes		Retest
	12c	**Adds sterile gel to outside of sheath on probe tip *or* directly onto patient**	Yes		Point lost
13. Adjust Patient Bed	13	**Sets bed to Trendelenburg position, and adjusts bed height if needed**	Yes		Point lost
14. Identify Target Vessel with Ultrasound	14a	**Verifies correct probe orientation**	No	Not part of AY16 training	Point lost
	14b	**Orients the probe correctly**	No	Poor US visibility in AY16 videos	Point lost
15. Anesthetize the Insertion Site	15	**Talks through anesthetizing the insertion site** *Note that we cannot actually inject with our simulators*	Yes		Point lost
16. Advance Seeker Needle Using	16a	**Positions needle and probe appropriately and maintains ultrasound image of vessel while advancing needle**	Yes		Retest

KU Health System CVC Insertion Procedural Steps		**Behavior to Demonstrate**	**Suitable for comparison**	**Reason if not comparable**	**Scoring rule**
Ultrasound and Aspiration	16b	**Grasps syringe suitably for aspiration while advancing**	Yes		Point lost
	16c	**Acquires an image of a hyperechoic spot on the ultrasound display, then pan back and forth to verify needle tip location**	No	Poor US visibility in AY16 videos	Point lost
	16d	**Acquires a "flash" of blue venous blood**	Yes		Retest
17. Remove Syringe	17	**Removes syringe from needle**	No	Frequent simulator errors in AY16	Point lost
18. Advance Guidewire	18	**Inserts guidewire 20-30cm at the skin**	Yes		Point lost
19. Confirm Guidewire with Ultrasound	19a	**Uses a longitudinal ultrasound view of the vessel area**	Yes		Point lost
	19b	**Acquires a view of the guidewire on the ultrasound display**	No	Poor US visibility in AY16 videos	Point lost
	19c	**Compressed the vessels with the probe**	No	Not part of AY16 training	Feedback
20. Nick Skin	20	*Note we cannot actually nick the simulator's skin*<br>**Demonstrates intended scalpel nick appropriately**	Yes		Point lost
21. Dilate Vein	21	**Advances the dilator approximately 3-4cm**	Yes		Point lost
22. Advance Catheter	22	**Inserts the catheter to 14-15cm at the skin**	Yes		Point lost
23. Remove Guidewire	23	**Removes the guidewire without letting go of it**	Yes		Point lost
24. Flush Ports	24	**Aspirates each lumen with caps on and with the syringe in an upright position, until blue blood is aspirated, then flushes each lumen**	Yes		Point lost
25. Secure and Dress Catheter	25a	**Places the Biopatch appropriately**	No	Not part of AY16 training	Point lost
	25b	**Places sutures appropriately**	Yes		Point lost
	25c	**Applies sterile dressing completely over the insertion site**	Yes		Point lost
26. Obtain Chest X-Ray	26	**Indicates that they would order a chest X-ray**	Yes		Point lost
27. Dispose of Sharps	27	**Indicates that they would dispose of sharps properly**	No	Not part of AY16 training	Point lost
28. Complete Procedure Promptly	28	**Completes procedure within 45 minutes**	No	Different number of items tested in AY16 & AY17	Incomplete steps lose points
